# Immune Responses Associated with Resistance to Haemonchosis in Sheep

**DOI:** 10.1155/2013/162158

**Published:** 2012-12-20

**Authors:** Fernando Alba-Hurtado, Marco Antonio Muñoz-Guzmán

**Affiliations:** Departamento de Ciencias Biológicas, Facultad de Estudios Superiores Cuautitlán, Universidad Nacional Autónoma de México, Km. 2.5 Carretera Cuautitlán-Teoloyucan, 54714 Cuautilán Izcalli, MEX, Mexico

## Abstract

This paper examines the known immunological and genetic factors associated with sheep resistance to infection by *Haemonchus contortus*. Such resistance is an inheritable genetic trait (*h* ^2^, 0.22–0.63) associated with certain sheep breeds. Resistant sheep do not completely reject the disease; they only harbor fewer parasites than susceptible sheep and therefore have a lower fecal egg count. Protective immune response to haemonchosis is an expression of genetic resistance. Genes associated with resistance and susceptibility are described. Genetically resistant sheep have nonspecific mechanisms that block the initial colonization by *Haemonchus contortus* larvae. These sheep also have an efficacious Th2 type response (e.g., increases in blood and tissue eosinophils, specific IgE class antibodies, mast cells, IL-5, IL-13, and TNF**α**) that protects them against the infection; in contrast, susceptible sheep do not efficiently establish this type of immune response. Finally, the main reported antigens of *H. contortus* were reviewed.

## 1. Introduction 

Gastroenteric verminosis is a disease with a great economic impact on sheep farms located in humid areas including tropical and subtropical regions of the world [1]. In Australia, losses due to this disease have been estimated at more than 400 million dollars (USD) per year; treatments in Kenya, South Africa, and India cost up to 26, 46, and 103 million USD, respectively [2]. Due to its ubiquity and virulence, *Haemonchus contortus* is the most important gastroenteric nematode of sheep in many regions of the world. It is a blood-sucking parasite of the abomasum that causes a disease known as haemonchosis [3, 4].

Haemonchosis is acquired by ingesting pasture contaminated with the third stage larvae (L3) of *H. contortus*. L3 penetrates the abomasal glands, where they molt into L4. The presence of larvae induces abomasal gland hyperplasia, inflammatory cell infiltration, and the substitution of wall cells secreting HCl with young nonsecreting cells. Consequently, the abomasal pH increases, which in turn reduces the transformation of pepsinogen to pepsin, reduces protein digestion, increases mucosa permeability, and increases the loss of endogenous proteins in the abomasum. Adult parasites are found in the abomasum lumen, and they are voracious hematophagous parasites, daily consuming 0.05 mL of host blood per worm [5]. The negative effects of haemonchosis on the biological and economic efficiency of sheep herds include malnutrition, low feed conversion, anemia, loss of appetite, low fertility indices, and in certain cases the death of young animals [3, 6]. 

Parasite control is based almost entirely on the administration of anthelmintic chemical compounds. Unfortunately, one of the problems generated by the massive, and indiscriminate use of anthelmintic products is the increasing resistance to these drugs, and this situation has huge consequences in those countries where sheep production is one of the main economic activities [7–9]. Together with the anthelmintic resistance problem, there is a trend toward the reduction of drug residues in human food and in the environment, which mandates that antiparasitic control strategies must not depend on chemicals. Among some of the proposed strategies are the development of specific vaccines against gastroenteric nematodes and the use of animal genotypes that are resistant to parasite infections. 

## 2. Resistance and Resilience

Nematode resistance includes the initiation and maintenance of a host response that prevents, reduces, or clears parasitic infection [10, 11]. Resistant animals do not completely reject the disease, but they have a lower parasitic load than susceptible animals, as measured by fewer eggs in their feces. This resistance is based on the immunological capabilities of each individual when challenged with parasitoses [12]. 

Resilience is the capacity of an animal to compensate for the negative effects of parasitism by the maintenance of productive parameters [13]. Sheep in general show simultaneously high resistance and resilience to haemonchosis. Some breeds have moderate or low resistance with relatively high resilience, allowing them to have productivity similar to those that are naturally resistant [14]. 

## 3. Breeds Susceptible and Resistant to Haemonchosis

 Differences between sheep breeds in their susceptibility to infection by abomasum-inhabiting nematodes were first reported by Stewart et al. [15], who described higher resistance to *Ostertagia circumcincta* (currently *Teladorsagia circumcincta*) in Romney Marsh lambs compared with lambs of the Rambouillet, Shropshire, Southdown, and Hampshire breeds and their crosses. Ross et al. [16] reported the first evidence for heritable resistance to haemonchosis in sheep. Subsequently, it has been shown that some sheep breeds are more resistant to gastroenteric nematodes than others.  Table 1 lists selected comparisons between breeds and the parameters of susceptibility or resistance that were measured. Additionally, there are individual differences within breeds [17]. 

 The resistance of some breeds can be explained by their place of origin. In general, resistant breeds were selected from areas where the climate favors the growth of gastroenteric nematode larvae in the environment, such that selection for certain productive parameters over several generations affected an indirect selection for nematode resistance. In fact, native breeds that have prospered despite unfavorable environmental conditions, poor zootechnical management, and no anthelmintic treatments are more resistant than highly productive breeds selected in areas with optimal health and zootechnical management [14]. 

 There are several ways to assess genetic resistance to gastroenteric nematodes. The most common method is the fecal egg count (FEC), which has intrinsic limitations because the number of eggs in feces is not necessarily correlated with the host's parasite load [18]. Low or reduced FEC has been used as a parameter for sheep selection in Australia [19, 20] and New Zealand [21]. The most trusted method to measure a sheep breed's resistance to gastroenteric nematodes is to count the total parasites (larvae and adults) in the gastrointestinal tract of the assessed sheep. Because this method can only be performed at necropsy, it is not useful for the genetic selection of sheep [10, 22].

 The use of haemonchosis-resistant sheep breeds has been proposed as a way to control the spread of drug-resistant strains of *H. contortus*. However, many of these breeds do not have the productive indices of other breeds; instead, some researchers are trying to select sheep for high resistance from productive breeds such as Merino and Romney, this resistance is a characteristic that is inherited by their descendants [19, 20]. The hereditability (*h* ^2^) of FEC varies between 0.22 and 0.63, indicating that selection for resistance or against susceptibility using this parameter can be moderately useful [23, 24]. Genetic markers associated with resistance could also be used to select sheep within a breed. There are many ongoing studies of resistance-associated genetic markers and some preliminary results. Alleles OMHC1-188 and OLADRB2-282 of the major histocompatibility complex (MHC) [25] and several quantitative trait loci (QTL) that contain diverse significant loci, such as the IFN*γ* locus in chromosome 3 [26, 27], have been associated with FEC reduction. Furthermore, some genes associated with the early inflammatory response including those encoding toll-like receptors (TLR2, 4 and 9) or involved with free radical production (DUOX1 and NOS2 A) are more abundantly expressed in lambs that are resistant to *H. contortus* and *Trichostrongylus colubriformis *infections [28].

## 4. Immune Response in Ovine Haemonchosis

The immunological mechanisms by which sheep have or acquire resistance to haemonchosis are not very clear [50, 53]; this resistance is an individual characteristic that has been associated with age, breed, and previous exposure to the parasite (infection or reinfection).

Both innate and adaptive immunities protect the host from *H. contortus* infection. Clearance of the nematode in immunized sheep requires several events, including the activation of nonspecific defense mechanisms, the recognition of parasitic somatic and excretion/secretion antigens, and the initiation of an appropriate acquired response [54].

### 4.1. Nonspecific Response Mechanisms to Haemonchosis

*H. contortus *larvae must inhabit an appropriate gastrointestinal niche that nourishes their development and growth and protects them from mechanical (peristaltic movement) and chemical (abomasum mucus) host barriers. Parasite colonization of the host abomasum initially depends on the motility of the larvae and the parasite load. Some host individuals, after sensitization via previous infections, can modify the microenvironmental conditions of the niche to expel the parasite [55].

Complement fixation is one of the first innate responses to *H. contortus *infection. Several studies demonstrated that helminths activate the alternate complement pathway and bind some molecules (opsonins) on their surface [56]. After larvae activate complement, vasoactive and chemotactic peptides (C3a and C5a) are generated, and these peptides mobilize eosinophils to the area of infection independently of specific mechanisms (CD4+ and IL-5). At the same time, *H. contortus* secretes chemoattractants for eosinophils and neutrophils, which reinforce the inflammatory response [57]. The thymus-independent increase in tissue eosinophils is an important innate response in which complement activation mediates the cytotoxicity of eosinophils against larvae in early infection stages in the absence of specific antibodies. 

When rodents are used as experimental models for gastrointestinal helminths, the quick elimination of parasites during the first infection is associated with inflammation induced by the alternate complement pathway and mediated by mast cells and eosinophils [56]. In contrast to rodent models, efficient elimination of nematode larvae in ruminants generally requires repeated infections [58].

Expulsion of *H. contortus* larvae in sheep can be immediate or delayed. Immediate expulsion occurs when larvae are attacked by tissue mast cells and a special type of intraepithelial mast cells (globule leucocytes) before the larvae enter their niche (abomasum gland). Similar to murine experimental models, other important mechanisms in the immediate expulsion from sheep are hypermotility, gastric hypersecretion, and hyperplasia of calciform cells with the subsequent increase in mucus production [55, 58]. These mechanisms may explain why some sheep breeds or genetically resistant genotypes counteract infection during its early stages.

 MacKinnon et al. [59] found that resistant and susceptible sheep breeds exhibited differential gene expression that was associated with an nonspecific response to *H. contortus*. At 3 days after infection (PI) with *H. contortus*, resistant sheep had reduced expression of genes associated with blood coagulation and higher expression of genes involved in the inhibition of coagulants, tissue repair and restructuring, blood vessel formation, and cell migration in the abomasum and abomasal lymph node. At day 27 PI, resistant sheep had higher expression of genes associated with intestinal motility, inflammatory response, cell differentiation and proliferation, and the reduction of apoptosis.

 Ghrelin is a growth hormone peptide (28 amino acids) of the stomach and is the endogenous ligand for GH secretagogue receptor [60]. It also stimulates appetite, regulates homeostasis of energy metabolism, and contributes to the modulation of the inflammatory response [61, 62]. Experimental infection with *H. contortus* in susceptible lambs reduces the expression of the ghrelin gene in abomasum and decreases the protein in plasma; in contrast, ghrelin gene expression and protein plasma content increase in resistant lambs [63]. Ghrelin reduction is most likely associated with appetite suppression and downregulation of the prolonged inflammatory response in susceptible lambs.

Immediate expulsion of the parasite is also associated with the presence of histamine and leukotrienes in the abomasum mucus, which inhibit the motility of nematode larvae *in vitro*. When challenged with the parasite, sheep immunized with *H. contortus* or *Trichostrongylus colubriformis* have a higher number of mast cells and globule leukocytes in the abomasum mucosa, and these cells have higher secretion of leukotrienes and factors that inhibit larvae migration [64]. High concentrations of histamine in the abomasal mucosa of sheep that are resistant to haemonchosis aid parasite expulsion by promoting abomasal hypersecretion and hypermotility, which are detrimental to the fecundity and motility of the worm [65]. Furthermore, histamine facilitates the translocation of plasma proteins including humoral antibodies into the lumen of the abomasum [55].

 Delayed expulsion of *H. contortus* larvae occurs when a specific immune response is mounted against the larvae in the abomasum glands. This action is regulated by CD4+ T lymphocytes, IgA and IgE antibodies, antibody-dependent eosinophil cytotoxicity, and the classic complement pathway [58]. 

 Tissue and blood eosinophils are increased during both the specific and nonspecific responses against gastrointestinal nematodes. The activation of the alternate pathway and degranulation of mast cells cause the increase and nonspecific degranulation of tissue eosinophils which is independent of IL-5. In addition to recruiting eosinophils to the abomasum wall, complement promotes eosinophil cytotoxicity against *H. contortus* larvae [66]. Infection with *Oestrus ovis* or inoculation with *Taenia hydatigena* larvae extracts induces eosinophilia in the abomasum and promotes resistance to haemonchosis in sheep [67, 68]. 

 Eosinophil degranulation releases major basic protein, cationic proteins, and peroxidase, which are cytotoxic to helminths. Lipid mediators such as leukotrienes, prostaglandin E2, platelet aggregation factor, and lipoxins are secreted, and these molecules promote increases in permeability, mucus secretion, chemotaxis, and coagulation. Eosinophils also produce cytokines IL4 and IL10, suggesting that these cells have a regulatory function in the immune response [69].

The exact role of *γδ* T lymphocytes is unclear. These cells have been associated with resistance to haemonchosis [4, 70], but it is unknown whether they are involved in resistance and/or immunity or if their presence is only a secondary effect *H. contortus *infection.

### 4.2. * H. contortus * Antigens 

 During the infection of sheep, *H. contortus *progresses through various life cycle stages (L3, L4, L5, and adult), among which there are differences in surface molecule expression. Some antigens specific to L3 and L4 are not expressed during the adult stage [71]. Quick changes in surface antigens make an effective adaptive response difficult in the initial stages of infection; therefore, each developmental stage is immunologically a different organism [54]. Thus, the larval antibody response does not cross-react with the adult stage. 

 Hidden antigens from the *H. contortus *intestine have been used to elicit a Th2-type response and the production of host serum antibodies, which are subsequently ingested when nematodes feed on the host's blood. The ingested antibodies recognize the nematode's intestinal antigens and alter its digestion [72]. The best-characterized and most effective intestinal antigens are the enzyme complexes H11 and H-gal-GP. The first is a family of microsomal aminopeptidases, and the latter is an aspartyl protease and metalloprotease complex. Together, these antigens, which have been obtained directly from adult worms, provide substantial protection against natural infection by *H. contortus* in sheep [73–75]. Immunization with H-gal-GP results in the production of host antibodies that inhibit the hemoglobinase activity of the endogenous enzyme, leading to *H. contortus *malnutrition due to decreased blood digestion [76]. However, the induced protection is short lived, and the difficulties of large-scale production of immunogens limit their commercial development. Sheep immunized with the same recombinant antigens expressed in *Escherichia coli* and insect intestinal cells have been unsuccessful to be protected from infection [75, 77]. 

 Other antigens have been evaluated as immunogens. Molina et al. [78] showed that immunization with cysteine protease-enriched protein fractions obtained from adult *H. contortus* worms protected sheep and goats against experimental infection with the parasite. The 70–83 kDa surface antigens obtained from exsheathed larvae, and the 15 and 24 kDa excretion/secretion antigens produce some degree of protection [79, 80]. Infection with different nematodes induces the abomasal and intestinal production of IgG antibodies against a carbohydrate larval antigen (CarLA) present on the surface of various strongylid nematodes. Incubation of exsheathed *Trichostrongylus colubriformis* larvae with these antibodies inhibited their implantation in the small intestine. However, the incubation of exsheathed *H. contortus* larvae with these antibodies did not have an effect on their implantation in abomasum [81].

 Haemonchosis resistance has been associated with alleles of the ovine MHC (OMHC1-188) and with certain surface molecules of ovine leukocytes (OLADRB2-282), suggesting that the mechanisms of antigen presentation differ between breeds [25, 82]. Some dendritic cells can internalize antigens homologous to those of *H. contortus *[83], so the specific response to *H. contortus* may be induced by the dendritic cell-mediated presentation of parasite antigens to helper T lymphocytes. Eosinophils also function as antigen-presenting cells, particularly in the case of helminthic infections. Eosinophils exposed to *Strongyloides stercoralis *antigens had increased expression of CD69, CD86, and MHC class II similar to dendritic cell controls, these eosinophils transformed *in vitro *naïve CD4+ lymphocytes to IL-5-producing CD4+ Th2 cells [84].

### 4.3. Antibodies and Resistance to Haemonchosis

Natural and experimental infections with *H. contortus* induce the production of specific antibodies. The serum antibody response has been widely studied although results have been variable. While some studies show an association between serum IgG levels and resistance [47], others found an association with infection but not with resistance [33, 45]. Abomasum antibodies are more important than serum antibodies in the protection against gastroenteric nematodes. High specific IgA levels in the abomasal mucus decrease the fertility and length of *Teladorsagia circumcincta*, which is another abomasum nematode of sheep [85]. There is a negative correlation between the amount of specific IgA in abomasum mucus and the parasite burden in *H. contortus *infections [45].

 A typical characteristic of helminthic infections is the induction of specific IgE, which results from a Th2-type response. IgE induces antibody-dependent cytotoxicity in eosinophils, mast cells, and macrophages. An increase in local IgE levels has been associated with resistance to gastroenteric nematodes in sheep and goats [86–88]. In *in vitro *assays, this immunoglobulin recognizes nematode surface allergens and directs eosinophils and mast cells to attack the parasite cuticle [48]. These functions are mediated by a high affinity IgE receptor present on the surface of these cells (Fc*ε*RI). A surface epitope of *H. contortus *has a *α*1→3-fuc domain that is recognized by IgE. This epitope was previously found in other helminths, plants, and some arthropods, and it has been associated with the induction of Th2-type responses and allergic processes [89]. 

Infection produces an increase in antibody-producing plasma cells, mainly of the IgA isotype [90]. Because the number of these cells is similar in susceptible and resistant sheep breeds experimentally infected with *H. contortus*, they have not been associated with resistance [91]. Activated B lymphocytes (CD45R+) are also increased during *H. contortus *infection [4, 58]. 

### 4.4. Immune Response Cells Associated with Resistance

Inoculation with *H. contortus* larvae induces T lymphocyte proliferation and the subsequent enlargement of abomasal lymph nodes and an increase in CD4+ lymphocytes in the abomasum wall and peripheral blood [70, 92, 93]. 

In experimental infections, CD4+ lymphocytes are required for inducing immunity in ovine haemonchosis. Neutralization of CD4+ lymphocytes by monoclonal antibodies negates *H. contortus *immunity and increases the parasite burden in sheep resistant to infection. This neutralization also suppresses mucosa mast cell hyperplasia, eosinophil infiltration of the abomasum, and the development of humoral memory response [94–96]. In contrast, the presence or absence of CD8+ lymphocytes seems to have no effect on resistance [58, 91].

Depending on the activation stimulus, murine helper CD4+ T lymphocytes differentiate into two cell types with different cytokine production profiles. Type 1 T lymphocytes (Th1), characterized by the production of IFN*γ* and IL-2 among others, constitute the cellular response and protect against intracellular parasites such as *Leishmania sp*. and *Toxoplasma gondii*. The Type 2 response (Th2), characterized by the production of IL-4, IL-5, and IL-10, is part of the humoral response and associated with the presence of helminths. The Th1 and Th2 responses are antagonistic to each other. The Th1 response inhibits the Th2 response through IL-10 [97]. The polarization of the Th1-Th2 response observed in mice and humans has not been demonstrated in ruminants, but it has been possible to establish the existence of a differentiated response associated with IL-5, eosinophils, mast cells, IgG1, and IgE in sheep resistant to haemonchosis [98]. There is also evidence that effector mechanisms of the Th2 type response are involved in immunity against *H. contortus* [50, 91].

It appears that susceptibility and resistance to haemonchosis depend on the type of immune response mounted against the parasite. CD4+ lymphocytes increase during experimental infection of both susceptible and resistant sheep. Thus, both groups respond to the presence of the parasite but do so in different manners. Compared with resistant sheep, susceptible sheep produce relatively more IFN*γ* and less parasite-specific serum antibodies, blood eosinophils, and abomasum eosinophils [98]; therefore susceptibility is most likely associated with a Th1 type response [47, 50], while resistance includes a Th2 type response. A differential response has also been observed in different abomasum regions. Muñoz-Guzmán et al. [91] found that resistant lambs experimentally infected with *H. contortus* had a Th2 type response (increase of eosinophils and CD4+ lymphocytes) in their abomasal pyloric region, and this response was not observed in the fundus region of the same lambs or in any abomasal regions of susceptible lambs. 

Other studies suggest that there is a Th1/Th2 dichotomy in sheep infected with gastroenteric nematodes. Gill et al. [98] studied the levels of IFN*γ* and IL-5 produced *in vitro* by abomasum lymphocytes stimulated with *H. contortus *antigens. Lymphocytes obtained from uninfected resistant sheep produced quantities of each cytokine similar to susceptible sheep, but lymphocytes obtained from infected resistant sheep produced less IFN*γ* and more IL-5 than lymphocytes obtained from susceptible sheep. These studies indicate that protection is mainly due to a Th2-type response.

Genetic studies confirm the aforementioned observations. Pernthaner et al. [99] showed that resistant sheep expresss the genes for IL-5, IL-13, and TNF*α* and do not express those of IL-4, IL-10, and IFN*γ*. Andronicos et al. [100] showed that, after the initial infection, there were no differences in cxcl10 gene (regulator of IFN*γ*) expression in the abomasum mucosa of lambs susceptible and resistant to haemonchosis. In subsequent infections susceptible lambs overexpressed this gene, which most likely made them incapable of establishing a protective Th2-type response. A similar effect was reported in mice, where overexpression of *cxcl10* decreased clearance of *Trichuris muris *infection in susceptible mice [101].

An essential factor modulating the type of response is the age at the time of infection. Lambs that are three to six months old have fewer CD4+ lymphocytes in the abomasum wall related to diminished immune response against *H. contortus *[102]. In contrast, a greater number of *γδ* T lymphocytes have been observed in the abomasum wall of young sheep [103]. Bovine *γδ* T lymphocytes stimulated with concanavalin A produced IL-2, IFN*γ*, and TNF*α* [104]. If the same pattern of cytokines is produced by *γδ* T lymphocytes of young sheep, they would mount Th1 type response. While this hypothesis could explain the high susceptibility of young lambs to infection, it requires the support of further studies.

In the first infection with *H. contortus*, the abomasum lymphocytes of susceptible sheep breeds do not produce cytokines associated with a Th2 response, but, in later infections, the production of these cytokines increases [58]. While these sheep do not reach the levels of resistance of genetically resistant sheep, the increased production of Th2 cytokines could contribute to the increased resistance to *H. contortus *in adult sheep of susceptible breeds.

## 5. Conclusions

 Resistance to haemonchosis is an inheritable genetic characteristic associated with some sheep breeds. The immune response that protects against *H. contortus* is the expression of this genetic resistance. Genetically resistant sheep have innate defense mechanisms that prevent their colonization by larvae during their first infection. Additionally, they establish a Th2 type immune response in the abomasum mucosa that protects them from infection, but susceptible sheep do not efficiently establish this type of immune response. Finally, the immune response and the associated resistance can be modified by the type of antigen that is recognized and by such factors as age, nutrition, and the number of infections.

## Figures and Tables

**Table 1:** Comparative studies of susceptibility/resistance to *Haemonchus contortus* or gastrointestinal nematodes between sheep breeds.

Resistant breed	Susceptible breed	Parasite	Infection	Evaluated parameters	References
Florida Native	Saint Croix and crossbred Dorset × Rambouillet	*H. contortus *	AI	Par, Hem, Immunol, Hist	[29]
Saint Croix	Dorper	*H. contortus *	NI	Par, Bioch, Immunol, Hist	[30]
Florida Native	Suffolk and Rambouillet	*H. contortus *	NI y AI	Par, Hem, Bioch, Immunol	[31, 32]
Red Maasai	Blackheaded Somali, Dorper and Romney Marsh	GINs	NI	Par, Hem	[1]
Castellana	Nil	*H. contortus *	AI	Par, Immunol	[33]
Florida Native and Pelibuey	—	GINs	NI	Par, Hem, LW	[34]
Rhön	Nil	*Trichostrongylus spp. *	NI	Par, Hem, Bioch	[35]
German Merino	Rhön	*H. contortus *	AI	Par, Hem, Immunol	[36]
Sabi	Dorper	GINs	NI	Par, Hem, LW	[37]
Blackbelly	INRA 401	*H. contortus* and *Trichostrongylus colubriformis *	AI	Par	[38]
Crossbred Saint Croix × Blackbelly	Crossbred				
	Dorset (50%),	GINs	AI y NI	Par	[6]
	Rambouillet (25%)				
	Finnsheep (25%)				
Katahdin and crossbred Blackbelly × Saint Croix	Dorper and Dorset	*H. contortus *	AI	Par, Hem, LW	[39]
Gulf Coast Native	Suffolk	GINs	NI	Par, Hem	[40]
Saint Croix	Katahdin and Dorper	GINs	NI y AI	Par, Hem, LW	[41]
Saint Croix	Katahdin and Suffolk	GINs	NI	Par, Hem, LW	[42]
Crioula Lanada of Brasil	Corriedale	*H. contortus *	NIT	Par, Bioch, Hem, Hist	[11]
Red Maasai	Dorper	*H. contortus *	AI	Par, Hem, LW, MP	[43]
Santa Ines	Ile de France	*H. contortus *	AI	Par, Hem, Bioch, Hist, Immunol, LW	[44]
Santa Ines	Suffolk and Ile de France	GINs	NI	Par, Hem, Hist, Immunol	[45]
Texel	Suffolk	GINs	NI	Par, Hist	[46]
Blackbelly	Columbia	*H. contortus *	AI	Par, Hem, Hist, Immunol	[47]
Blackbelly	INRA 401	*H. contortus *	AI	Par, Hist, Immunol	[48]
Lohi	Thalli and Kachhi	*H. contortus *	AI y NI	Par, Hem, Bioch, Hist, LW	[49]
Criolla Native to the Central Mexican Plateau	Suffolk	*H. contortus *	AI	Par, Hem, FT, LW	[14]
Gulf Coast Native	Suffolk	*H. contortus *	AI	Par, Immunol	[50]
Santa Ines	Ile de France	*H. contortus *	AI	Par. Immunol	[51]
Canaria Hair	Canaria	*H. contortus *	AI	Par.	[52]

NI: natural infection; AI: artificial infection; GINs: gastrointestinal nematodes; Par: parasitological (fecal eggs count, worm burden, etc.); Hem: hematological (packed cell volume, blood eosinophils, etc.); Immunol: immunological (antibodies); Hist: cellular count in abomasum (eosinophils, leucocytes, mast cells, etc.); Bioch: biochemical (serum protein, albumin, etc); LW: live weight; MP: mortality percentage FT: FAMACHA test.
